# Follicle dynamics: visualization and analysis of follicle growth and maturation using murine ovarian tissue culture

**DOI:** 10.1007/s10815-017-1073-5

**Published:** 2017-10-27

**Authors:** Tomohiko Murase, Akira Iwase, Kouji Komatsu, Bayasula, Tomoko Nakamura, Satoko Osuka, Sachiko Takikawa, Maki Goto, Tomomi Kotani, Fumitaka Kikkawa

**Affiliations:** 1 Department of Obstetrics and Gynecology, Nagoya University Graduate School of Medicine, 65 Tsurumai-cho, Showa-ku, Nagoya, 466-8550 Japan; 2 Department of Maternal and Perinatal Medicine, Nagoya University Hospital, 65 Tsurumai-cho, Showa-ku, Nagoya, 466-8550 Japan; 3 Department of Physiology, Aichi Medical University, 1-1 Yazakokarimata, Nagakute, Aichi 480-1195 Japan

**Keywords:** Follicular development, Granulosa cells, Oocyte maturation, Ovary, Time-lapse imaging

## Abstract

**Purpose:**

To visualize and analyze follicle development in ovarian tissue culture using physiological concentrations of follicle-stimulating hormone (FSH) and luteinizing hormone (LH) in order to establish an ovarian tissue culture system that enables efficient in vitro growth of follicles.

**Methods:**

Ovarian tissues from 4-week-old female ICR mice were sliced and cultured. Images of ovarian tissues in culture were obtained at 24-h or 30-min intervals by using a microscope. The area of each follicle observed in the ovarian tissue slices was tracked and analyzed in association with oocyte maturation.

**Results:**

We were able to track the development of each follicle using this culture system. Follicle growth was associated with oocyte maturation. Meiotically matured oocytes (MII) were obtained from 33% of all follicles investigated. Approximately, a quarter of follicles (24%) did not grow and resulted in atresia.

**Conclusion:**

Follicle dynamics were successfully visualized and analyzed in murine ovarian tissue culture. We were able to obtain mature oocytes from the fully grown follicles in vitro. This culture system would be helpful for efficient in vitro culturing of ovarian tissues.

**Electronic supplementary material:**

The online version of this article (10.1007/s10815-017-1073-5) contains supplementary material, which is available to authorized users.

## Introduction

A follicle in mammalian ovaries is a basic functional unit. Primordial follicles are recruited and grow to primary, secondary, and antral-stage follicles. Follicle development is generally classified into gonadotropin-independent and gonadotropin-dependent stages, according to the response to gonadotropins [1]. Gonadotropins, such as follicle-stimulating hormone (FSH) and luteinizing hormone (LH), are significantly involved in the gonadotropin-dependent stage in an endocrine manner. On the other hand, follicle development in the gonadotropin-independent stage is regulated with intra-ovarian growth factors, such as growth differentiation factor-9, in an autocrine and paracrine manner [2, 3].

Folliculogenesis has been evaluated by counting the number of follicles in various developmental stages including primordial, primary, and secondary follicles. This technique, known as “follicle dynamics analysis,” has been applied to assess the induction and suppression of folliculogenesis both in in vivo and in vitro experiments [4, 5]. However, to our best knowledge, follicle dynamics has never been visualized in real time.

A follicle consists of the oocyte and two types of somatic cells surrounding the oocyte, the granulosa and theca cells, which play principal roles in steroidogenesis. Granulosa cells proliferate and gain capacity for steroidogenesis in response to several stimuli, such as FSH and LH, and therefore play significant roles in follicular development [6]. The apoptosis of granulosa cells results in follicular atresia due to the insufficiency of survival signals and/or physiological/non-physiological apoptotic signals [7, 8]. Taken together, healthy folliculogenesis and steroidogenesis requires normal proliferation of granulosa cells depending on the specific developmental stage of the follicles.

In the current study, we analyzed follicle development in sliced murine ovarian tissue under culture conditions in the presence of FSH and LH using a time-lapse imaging system. We tracked the size of each follicular area and assessed the follicle dynamics visualized in this culture system. These novel analytical methods revealed useful information on follicle dynamics and proliferation of granulosa cells in in vitro ovarian tissue culture that might be necessary to complete in vitro growth of follicles from primordial to mature Graafian follicles.

## Materials and methods

### Analysis of follicle dynamics visualized in the ovarian tissue culture

**Fig. 1:**
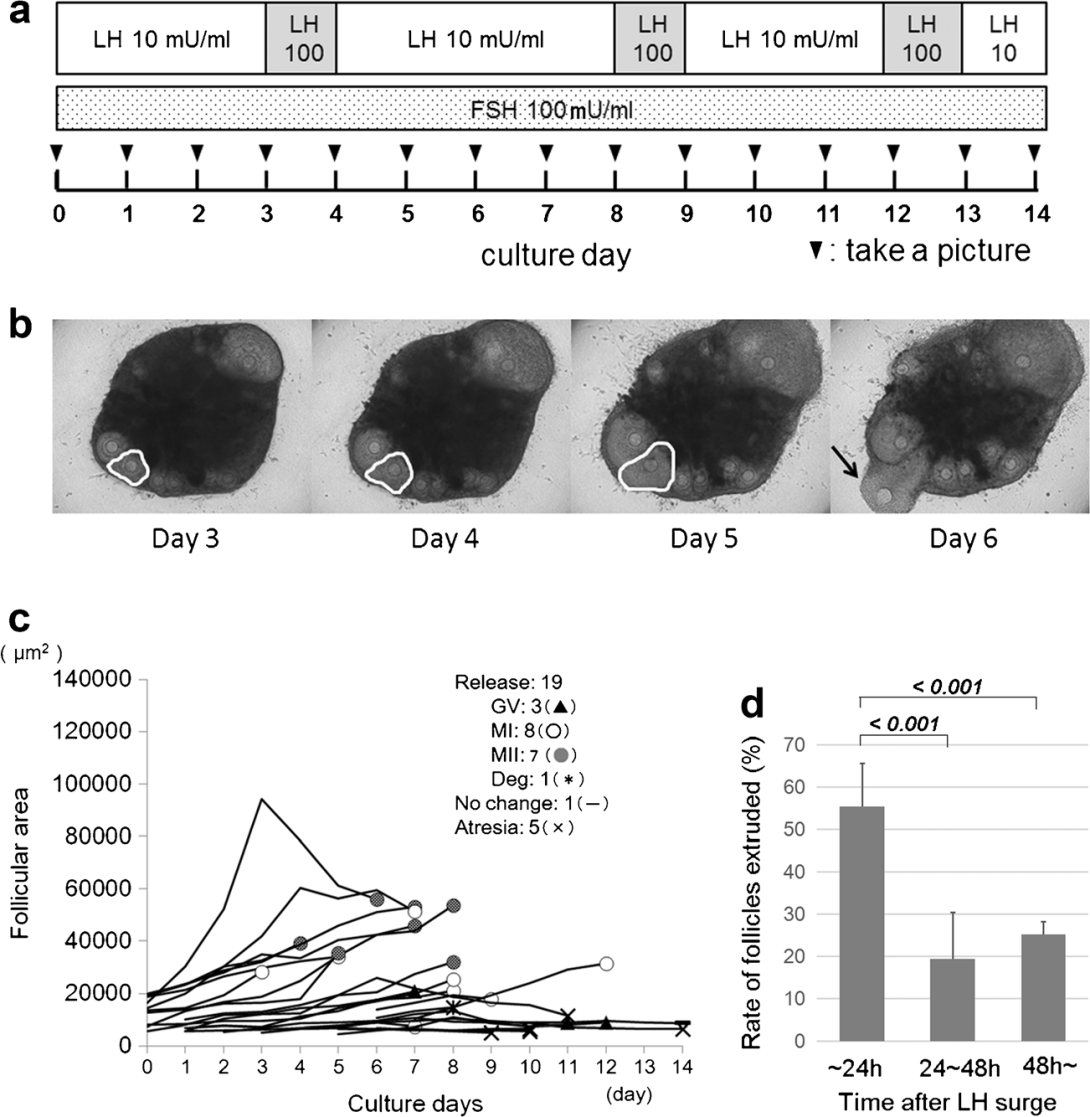
Evaluation of follicle growth in ovarian tissue culture. **a** Culture condition of murine ovarian slices for 14 days with physiological concentration of constant FSH and LH with periodic surge-like loading. **b** Outlined areas were determined as follicular area of growing follicles. Some oocytes were extruded from the ovary during culture (arrow). **c** Sizes of each follicle during in vitro culture are shown. The numbers of released oocytes of each meiotic phase (GV, MI, MII, or degeneration) are embedded in the panel. Similar results were obtained from three independent experiments. The graph showed the representative data. **d** Approximately 55.4% follicle extrusion occurred within 24 h after each LH surge. Italicized numbers indicate the *P* values calculated using one-way ANOVA with Bonferroni’s pairwise comparison. All values are indicated as the mean ± SD

Ovaries were resected from 4-week-old female ICR mice (Charles River Laboratories Japan, Inc., Yokohama, Japan) and cut into four equal slices, approximately 500 μm each in thickness, using a microtome blade under a stereoscopic microscope. We analyzed 12 ovarian tissue slices derived from three ovaries of three mice. The ovarian tissue slices were cultured using a 30-mm cell culture insert (Merck KGaA, Darmstadt, Germany) in Minimum Essential Medium Alpha GlutaMax (Thermo Fisher Scientific Inc., Waltham, MA, USA) supplemented with 5% (*v*/*v*) fetal bovine serum (Biological Industries Ltd., Beit HaEmek, Israel), 100 mIU/mL FSH, and 10 mIU/mL LH (Merck KGaA) at 37 °C in 5% CO_2_ atmosphere for 2 weeks. The culture conditions have been illustrated in Fig. 1a. The concentrations of FSH and LH were determined as reported previously [9]. A tenfold periodic increase in LH concentration was induced for surge-like loading. Therefore, the culture medium was changed every day. Follicle development in the four slices of ovarian tissues was simultaneously tracked. Images of ovarian tissues in culture were obtained at 24-h intervals by using a confocal microscope (FV1000; Olympus, Tokyo, Japan). The area of each follicle observed in ovarian tissue slices was measured using the ImageJ software (National Institutes of Health, Bethesda, MD, USA). The outlines of the follicles in the captured bright-field images were traced with a tablet pen (Intuos; Wacom Co., Ltd., Kazo, Japan), and the number of pixels in each follicle was measured. The pixel counts were converted to areas measured in square micrometers. The follicle areas were tracked as the follicles grew. Follicles with an intact basement membrane and a dark oocyte or absent delineation of the oocyte membrane were classified as atretic follicles [10].

Extruded oocytes were isolated and the meiotic phase of the oocytes was determined. Oocytes were considered arrested at prophase I in the germinal vesicle (GV) stage if the nucleus was intact, but were classified as belonging to metaphase I (MI) if the nucleus was not visible. If a polar body was present in the perivitelline space, the oocytes were classified as belonging to metaphase II (MII). Fragmented or shrunken oocytes were classified as degenerated (D). Time-lapse images of ovarian tissue slices cultured in a similar manner were obtained using the BZ-X700 fluorescence microscope (Keyence, Osaka, Japan) every 30 min.

### Statistical analyses

All data were analyzed using the SigmaPlot13 software program (Systat Software Inc., San Jose, CA, USA). Simple linear regression analysis and the Pearson correlation were applied to compare oocyte diameters and follicular areas. One-way ANOVA with Bonferroni’s pairwise multiple comparison test was used to compare the follicular extrusion rates between each time interval after LH surge and follicular areas between oocyte maturation stages. A *P* value <0.05 was considered to be statistically significant.

## Results

To evaluate follicle development in the ovarian tissue culture system, we extended the culture period for 2 weeks (Fig. 1a). We identified the proliferation of granulosa cells and development of follicles in this system. The area of each follicle to be measured was clearly identified (Fig. 1b). We analyzed each follicle development using this visualized culture system (Fig. 1c). The number of follicles tracked in each tissue slice ranged from 2 to 10. The average number and SEM of follicles was observed to be 7.08 ± 0.85. The total number of tracked follicles in each ovary was 25, 30, and 34. We observed that a significantly larger number of oocytes extruded within 24 h after each LH surge (Fig. 1d). Some of the growing follicles resulted in extrusion of oocytes. Oocyte maturation at the time of extrusion varied from GV to MII. Other follicles did not grow and turned out to have atresia. The number of extruded oocytes in each meiotic phase and atretic follicle has been shown in Table 1. The meiotically matured oocyte (MII) was limited to 33% of all oocytes/follicles investigated in this culture system. In addition, approximately a quarter of follicles (24%) did not grow and resulted in atresia.

**Table 1:** Number of extruded oocytes in each meiotic phase and atretic follicle

	1st	2nd	3rd	Total
Release				
GV	3	9	3	15 (17%)
MI	8	5	9	22 (25%)
MII	7	11	11	29 (33%)
Degenerated	1	0	0	1 (1%)
Atretic follicle	5	5	11	21 (24%)
Total	24	30	34	88 (100%)

**Fig. 2:**
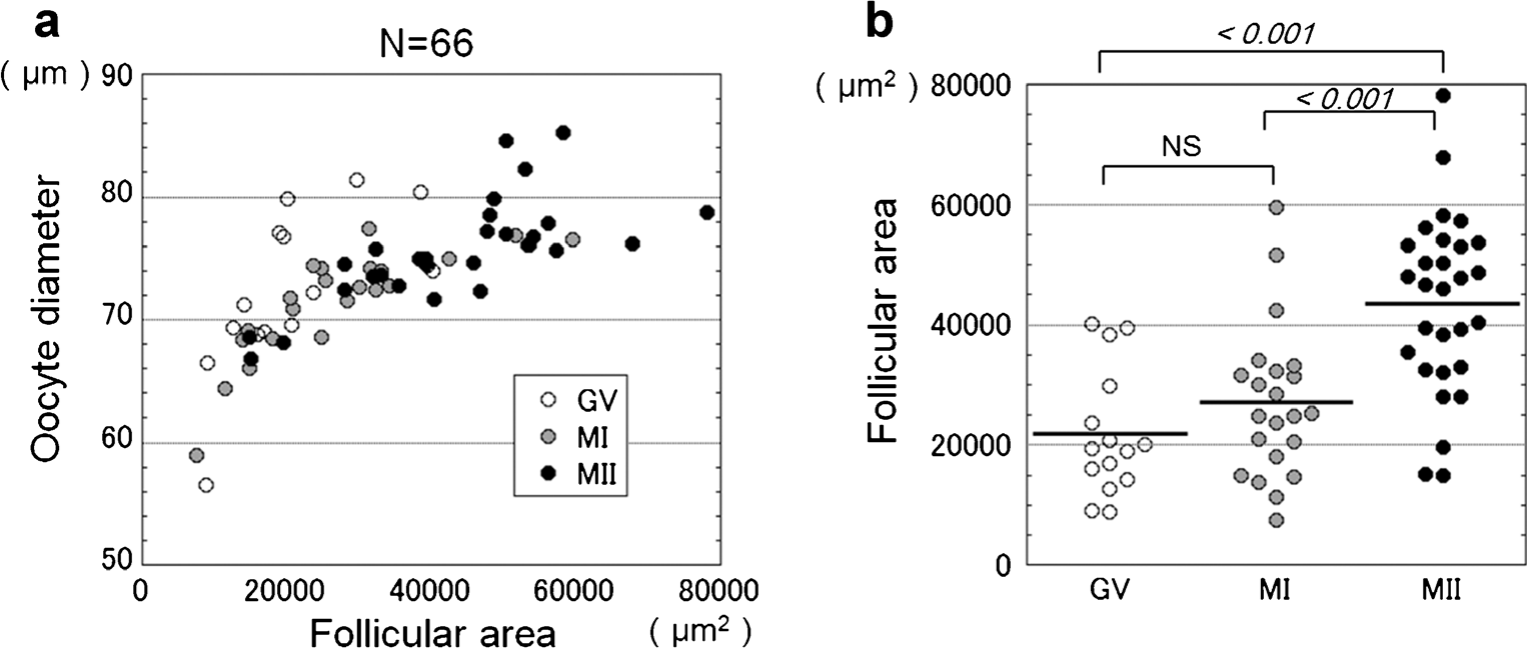
Correlation of follicular area, diameter and meiotic stage of oocytes. **a** Oocyte diameter was positively correlated with follicular area in ovarian tissue culture (*r* = 0.714, *P* < 0.001). **b** Meiotic maturation of oocytes was correlated with follicular area. Follicular area of released oocytes was significantly different between GV and MII, and MI and MII. Italicized numbers show *P* values in One-way ANOVA with Bonferroni’s pairwise comparison. NS, no significance. Bar, median

Next, we analyzed the correlation between follicular area, oocyte diameter, and the meiotic stage when the oocytes were extruded. We found that oocyte diameter was correlated with follicular area and that the follicle sizes increased according to the stage of meiosis (Fig. 2).

Further, we tried much more frequent manipulation of images using a built-in microscope incubator that enabled us to create time-lapse cinematography for follicle development, as shown in the supplemental data (Supplemental Video 1). Similarly, as shown in Fig. 1c, most follicles grew in size in conjunction with granulosa cell proliferation and resulted in the extrusion of oocytes at various maturation stages.

## Discussion

In the present study, we established the visualization of follicle development using sliced ovarian tissue culture so that we could trace the follicle areas and track follicle development. Histological analysis using fixed ovarian tissues has been widely used to confirm possible follicle development in ovarian tissue culture [11, 12]. However, using this method, it is impossible to obtain detailed information about which follicles, and when and how they are growing. Our system has made it possible to analyze the movement of a follicle cohort in the ovary, which is called “follicle dynamics.” In the current study, follicle growth was mainly evaluated using manual inspection once a day. It was possible to clearly track the sizes of oocytes and follicles and confirm a good correlation between these two parameters during follicle development in ovarian tissue culture. It was also found that as follicles grew, more oocytes were likely to mature. These findings suggest that this system maintains the synchronicity between follicle growth and oocyte maturation observed in vivo. Moreover, a built-in microscope incubator enabled us to create time-lapse cinematography for follicle development, as shown in the supplemental data (Supplemental Video S1). This technique of time-lapse cinematography has immensely contributed to the visualization and evaluation of embryo development [13]. We were the first to report the safety of this technique [14]. Detailed analysis using time-lapse cinematography in ovarian tissue culture could be useful for better understanding of folliculogenesis.

Histological counting of the number of follicles at each stage has been used to determine whether certain molecules, such as members of the transforming growth factor-β superfamily, exert effects on follicle development. However, the process of recruitment and induction of follicle development is rather complex, whereby several molecules are interacting mutually in a bidirectional manner between oocytes and somatic cells, including the granulosa and theca cells. Therefore, simply counting the number of follicles in a specimen cannot determine the specific effects of stimulants used in vitro and in vivo on follicle development.

The goal of ovarian tissue culture is to gain a better understanding of folliculogenesis and the establishment of complete in vitro follicle growth from primordial follicles. We believe that visualization of follicle development using our system will be useful for evaluating the mechanism(s) and molecules that regulate folliculogenesis in vitro. Therefore, it would be helpful in determining the optimal conditions required for in vitro culturing of ovarian tissues. Treatments for malignant diseases, such as radiation and chemotherapy, pose a risk for gonad toxicity and may affect fertility in future. Ovarian insufficiency is the most frequent cause of iatrogenic infertility. Ovarian tissue cryopreservation followed by autologous transplantation is currently the only available option for these patients and this technique is now becoming widespread [15]. On the other hand, transplantation of cryopreserved ovaries might cause the reintroduction of malignant cells concealed in these ovaries [16, 17]. The technique demonstrated in the present study might eliminate the procedure of transplantation of cryopreserved ovaries and the risk of reintroducing malignant cells in these ovaries. A multistep culturing system combining ovarian tissue culture and follicle culture from late secondary follicles has been established in mice, although its efficiency is not very high [18–20]. Compared to mice, the period from primordial to late secondary follicles is much longer in humans. Therefore, an ovarian tissue culture system preceding the follicle culture would be more important and require longer period.

One of the limitations of the current study is efficiency of oocyte maturation. Our culture medium containing physiological concentrations of FSH and LH with periodical surge-like concentration contributed to follicle growth and oocyte maturation. However, the rate of MII oocytes was approximately less than 40%. One of the possible reasons may be that the time required for oocyte extrusion in the sliced ovarian tissues could be shorter compared to the time required for ovulation. Lower maturation rates of the extruded oocytes could be attributed to their non-physiological premature extrusion from the follicles. In vitro maturation following ovarian tissue culture might be helpful for GV oocytes to develop into MII oocytes. Extrusion of oocytes followed proliferation of the granulosa cells. In addition, we confirmed that the extruded mature oocytes possessed fertilization capacity (unpublished data). However, there may be several differences between physiological ovulation and the extrusion of oocytes observed in our system. One of the reasons is the weakness of follicle structure caused by slicing the ovarian tissue. Three-dimensional in vitro culturing using alginate gel might be helpful in resolving this problem. Moreover, some follicles did not grow and resulted in atresia. In the current system, murine ovarian tissues were sliced and cultured so that nutritional factors could infiltrate into them. Therefore, on contrary, intra-ovarian growth factors that may help in follicle growth in an autocrine and paracrine manner would be possibly dispersed into the medium. Exogenous administration of these factors may be helpful to induce more efficient follicle growth. Further study will be needed to prove this hypothesis.

Another limitation of our system is the difficulty in identification of the primordial follicles. Recruitment of primordial follicles into growing follicle cohort is the initial and an important step in follicle development. Further improvement is required for the identification and visualization of primordial follicle recruitment. Our culture system is potentially compatible with the conventional histological methods for calculating the number of follicles in each stage.

In this study, we established a sliced ovarian tissue culture system that is capable of directly evaluating follicle dynamics in vitro. This culture system may be helpful for the determination of suitable conditions for induction and suppression of folliculogenesis in ovarian tissue culture.

## Electronic supplementary material

Supplemental Video 1 Time-lapse cinematography of ovarian tissue slice culture. (MP4 33,392 kb)

ESM 2 (GIF 419 kb)

High resolution image (TIFF 5439 kb)
