# Supplementary figures and images for: Follicle dynamics: visualization and analysis of follicle growth and maturation using murine ovarian tissue culture
Source: J Assist Reprod Genet. 2017 Oct 27;35(2):339–43. doi: 10.1007/s10815-017-1073-5 (PMC5845041; doi:10.1007/s10815-017-1073-5)

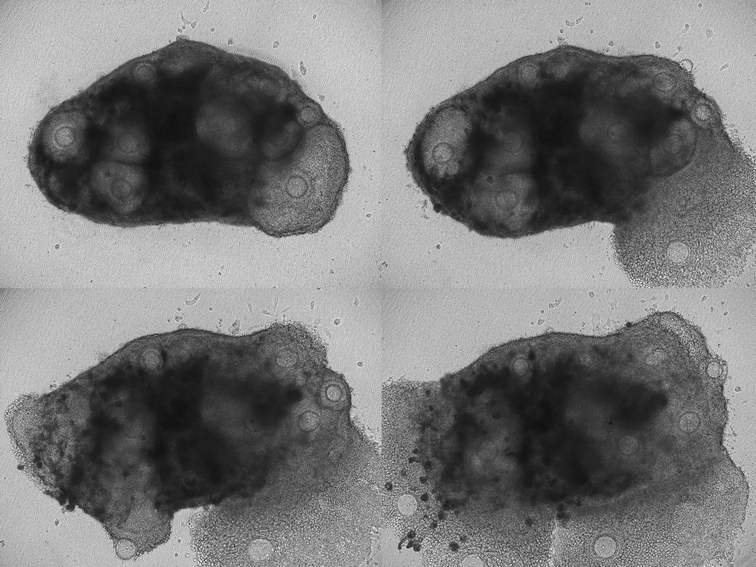

Supplement: Supplementary file 2 — (GIF 419 kb) [file 10815_2017_1073_Fig3_ESM.gif]

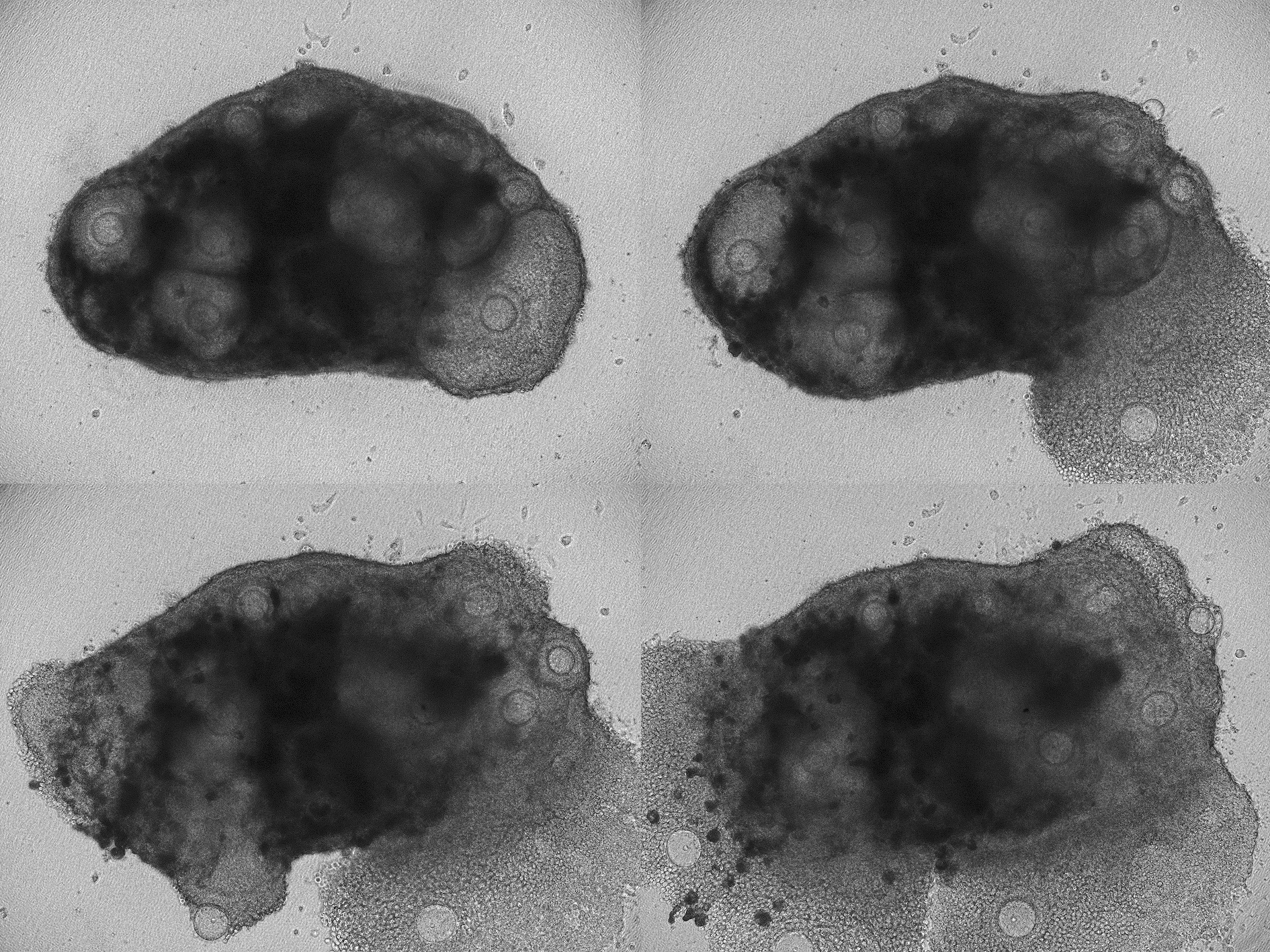

Supplement: Supplementary file 3 — High resolution image (TIFF 5439 kb) [file 10815_2017_1073_MOESM2_ESM.tif]
